# A new species of the genus *Ochthebius* (Coleoptera, Hydraenidae) from the Ogasawara Islands, Japan, with a description of the larva

**DOI:** 10.3897/zookeys.855.35677

**Published:** 2019-06-13

**Authors:** Hiroyuki Yoshitomi, Haruki Karube, Masakazu Hayashi

**Affiliations:** 1 Entomological Laboratory, Faculty of Agriculture, Ehime University, Tarumi 3-5-7, Matsuyama, 790-8566, Japan Ehime University Matsuyama Japan; 2 Kanagawa Prefectural Museum of Natural History, Odawara, Iryuda 499, Odawara, Kanagawa 250-0031, Japan Kanagawa Prefectural Museum of Natural History Kanagawa Japan; 3 Hoshizaki Green Foundation, Sono, Izumo, 691-0076, Japan Hoshizaki Green Foundation Izumo Japan

**Keywords:** Ochthebiinae, Ochthebiini, Bonin Islands, chaetotaxy, Staphylinoidea

## Abstract

A new species of the genus *Ochthebius*, O. (O.) sasakii**sp. nov.**, is described from the Ogasawara Islands, Japan, with a description of the larva. This record is the first of the family Hydraenidae from the Ogasawara Islands. This species belongs to the *punctatus* species group and is similar to two Japanese species, O. (O.) inermis Sharp, 1884 and O. (O.) danjo Nakane, 1990, but it differs from them in both adult and larval characters. The larva of O. (O.) inermis is also described for comparison.

## Introduction

The Ogasawara Islands (= Bonin Islands) are oceanic islands that were never connected to the continental mainland (Japan Wildlife Research Center 2010); therefore, the islands contain many endemic species within a small geographic area and are registered as a UNESCO World Heritage Site (WHS). The fauna of the islands is comparatively well studied (Ohbayashi et al. 2004), and the coleopteran fauna was reviewed by Kurosawa (1976a, b). A few aquatic beetles were recorded from the islands (Ohbayashi et al. 2004); the only known endemic species is *Copelatusogasawarensis* Kamiya, 1932 (Dytiscidae, Copelatinae).

The genus *Ochthebius* Leach, 1815 (Ochthebiinae, Ochthebiini) is distributed in the Palaearctic, Oriental, Nearctic, Neotropical, Afrotropical, and Australian regions, and includes 540 species within 10 subgenera (Villastrigo et al. 2019). Fourteen species of the genus *Ochthebius* are recorded from Japan under two subgenera and five species groups (Yoshitomi and Satô 2001; Villastrigo et al. 2019), but no species are recorded from the Ogasawara Islands.

In the present paper, we describe a new species of the genus *Ochthebius* from the Ogasawara Islands, with a description of the larva. This record is the first of the family Hydraenidae from the Ogasawara Islands. The larva of *O.inermis* Sharp, 1884 is also described for comparison with the larvae of the new species and *O.danjo* Nakane, 1990.

## Material and methods

The material examined in this paper is preserved in the Ehime University Museum, Matsuyama, Japan (EUMJ); Kanagawa Prefectural Museum of Natural History, Odawara (KPMNH); and Naturhistorisches Museum Wien (NMW).

General observations, dissections, and microstructures of dissected parts were made under a Leica MZ95. After observation, the dissected parts were mounted on the same card with the specimen. Photographs were taken under a Leica MZ95.

The terminology follows Jäch et al. (2016) for adults, and Delgado and Soler (1997), Delgado and Matsui (2000), and Delgado (2003) for larval chaetotaxy.

Morphological abbreviations used in this study are as follows:

**EL** elytral length from anterior margin to elytral apex;

**EW** maximum elytral width;

**HL** head length;

**PL** pronotal length in median line;

**PW** maximum width of pronotum;

**TL** total length (PL + EL + HL).

The average is given in parentheses after the range.

## Taxonomy

### Ochthebius (Ochthebius) sasakii sp. nov.

Taxon classificationAnimaliaColeopteraHydraenidae

http://zoobank.org/7911B6EA-6485-4D6E-80E0-3B65107A4083

**Figure 1.**
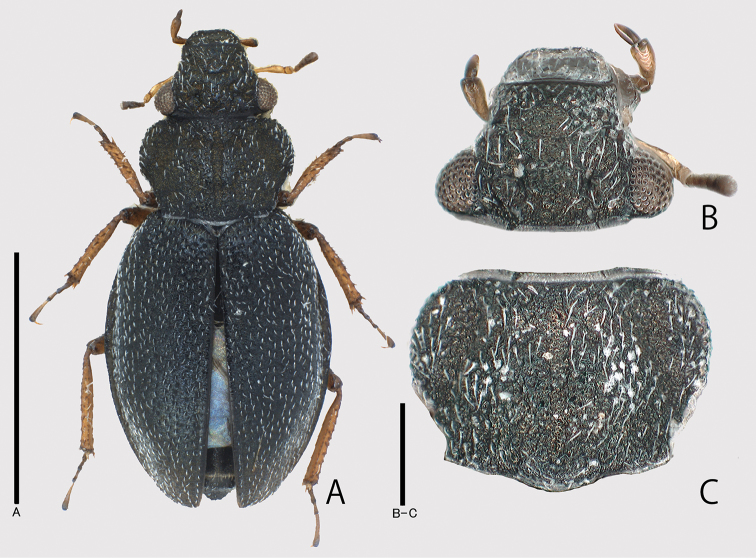
Holotype of *Ochthebiussasakii* sp. n. **A** dorsal habitus **B** head **C** pronotum. Scale bars: 1.0 mm.

**Figure 2.**
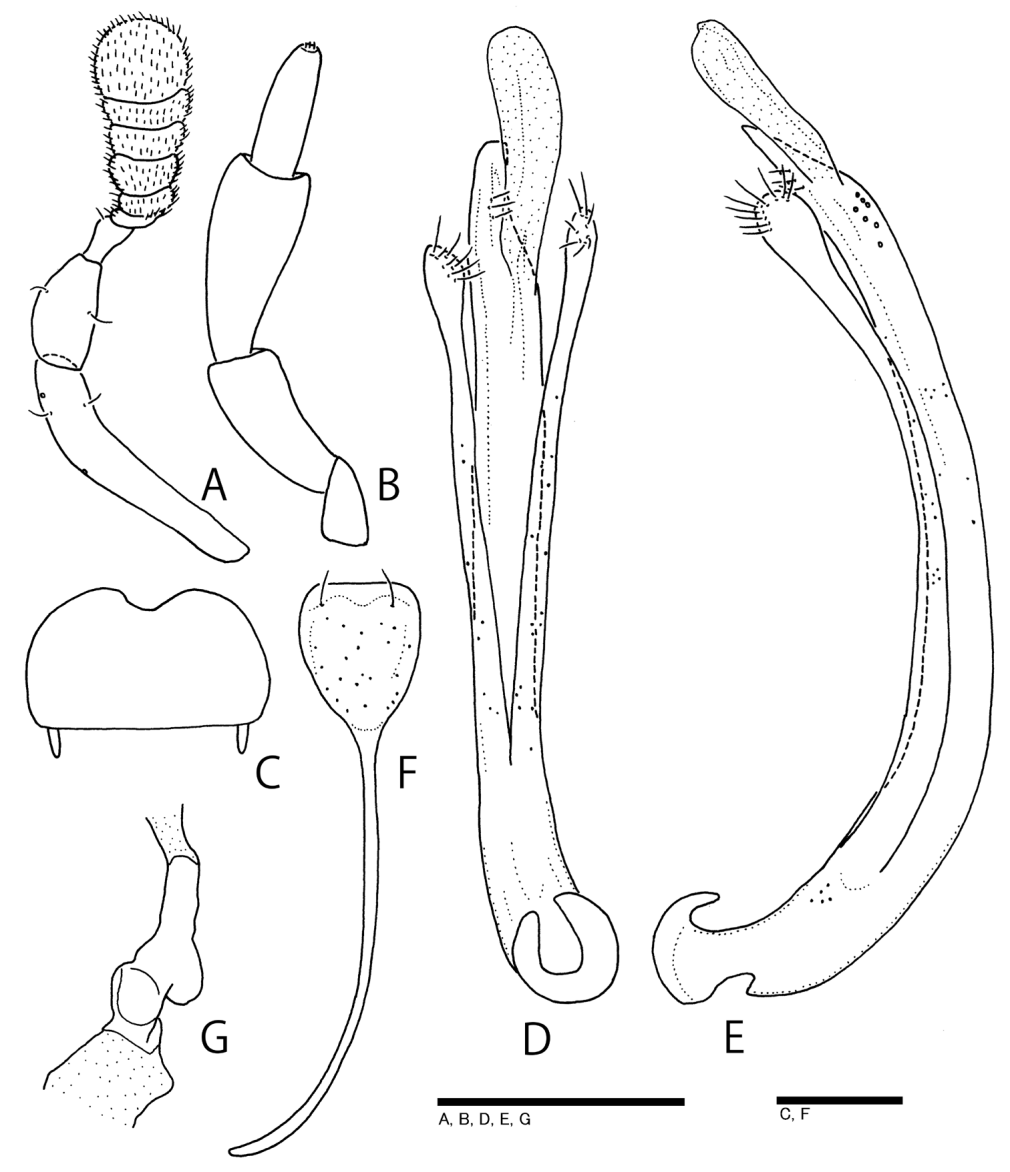
*Ochthebiussasakii* sp. n., paratypes, male (**A–F**) and female (**G**). **A** antenna **B** maxillary palpus **C** labrum **D** aedeagus in ventral view **E** aedeagus in lateral view **F** sternite X and spiculum gastrale **G** spermathecal duct. Scale bars: 0.1 mm.

**Figure 3.**
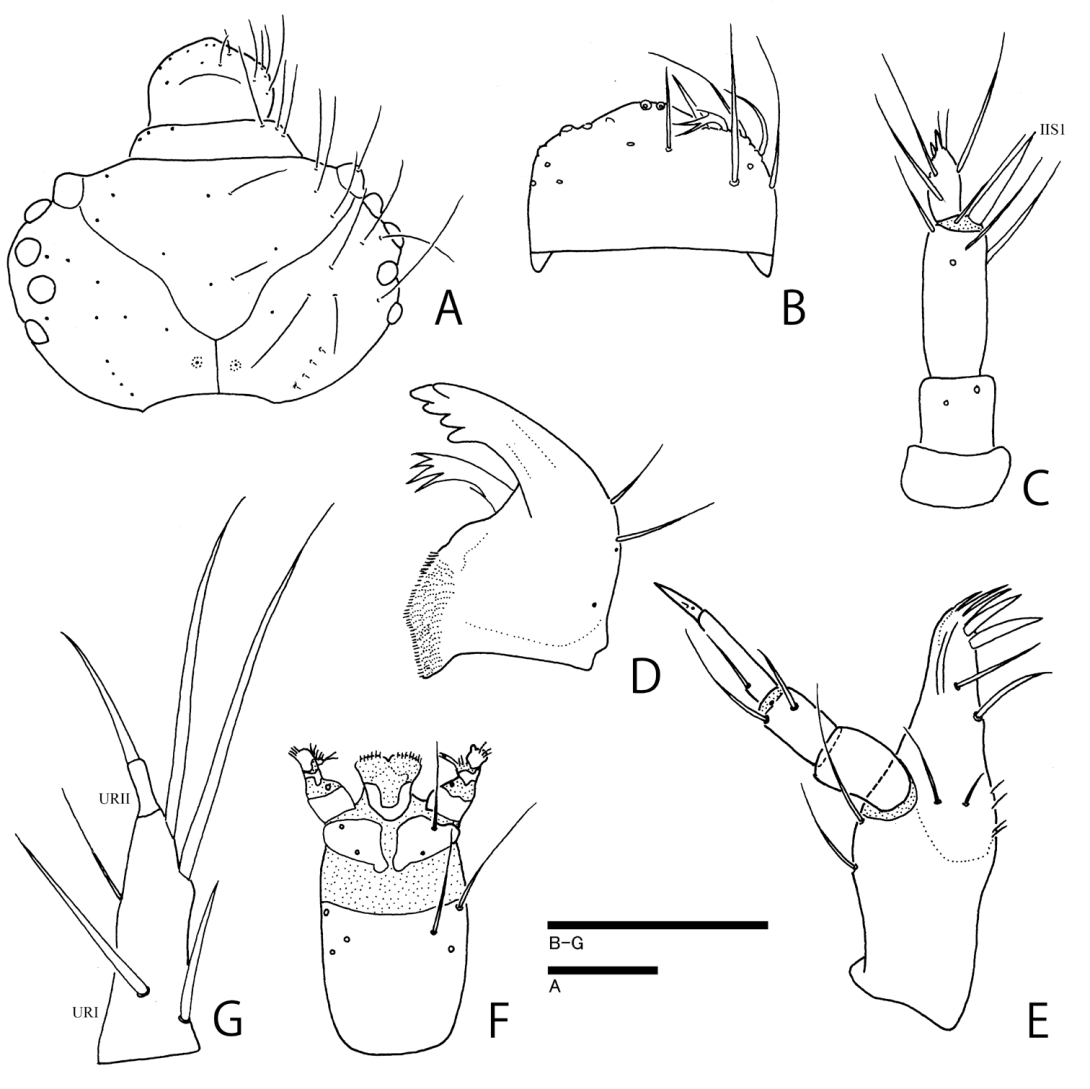
Larva of *Ochthebiussasakii* sp. n. **A** head capsule **B** labrum **C** antenna **D** mandible **E** maxilla **F** labium **G** urogomphus. Scale bars: 0.1 mm.

**Figure 4.**
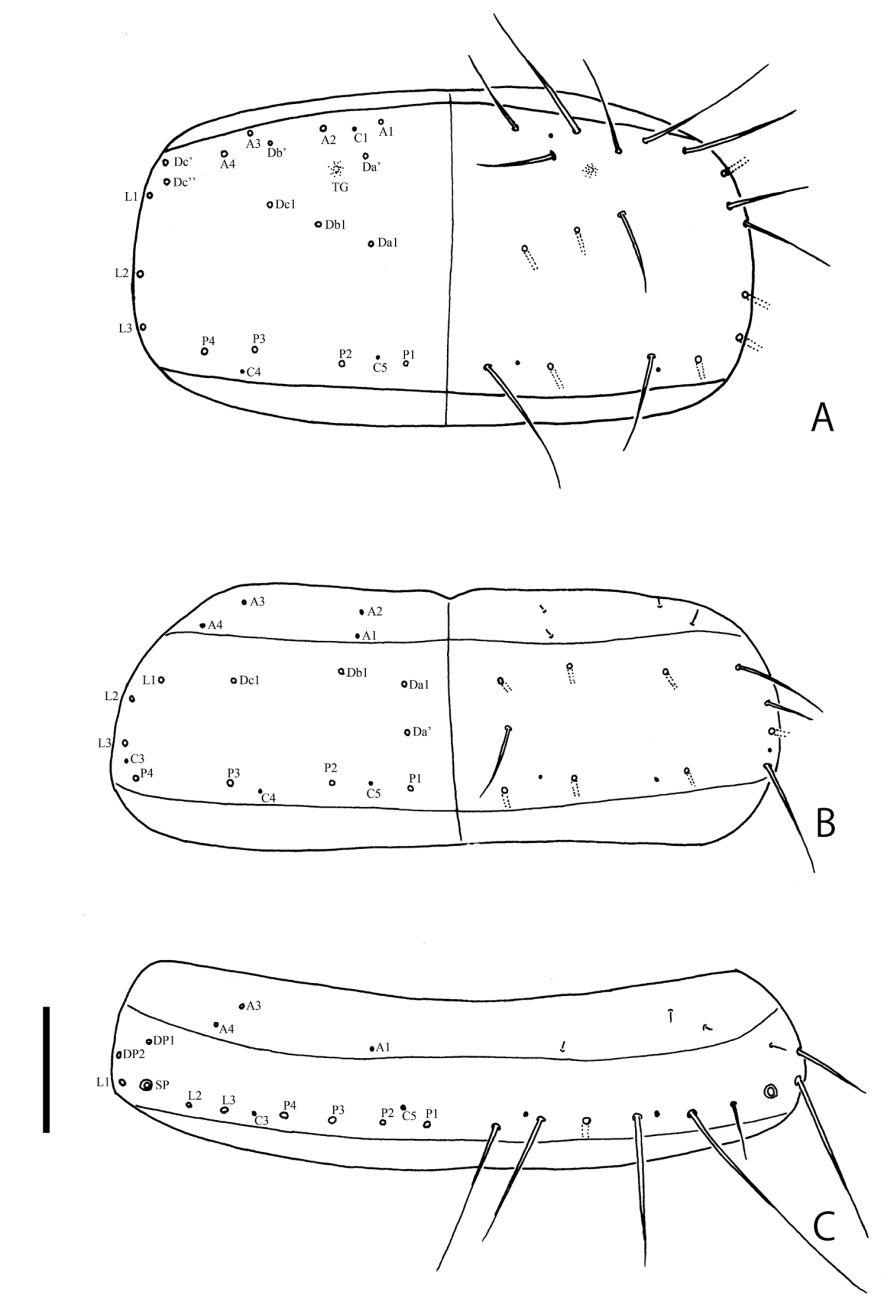
Larval chaetotaxy of *Ochthebiussasakii* sp. n. **A** pronotum **B** mesonotum **C** abdominal tergum I. Scale bar: 0.1 mm.

**Figure 5.**
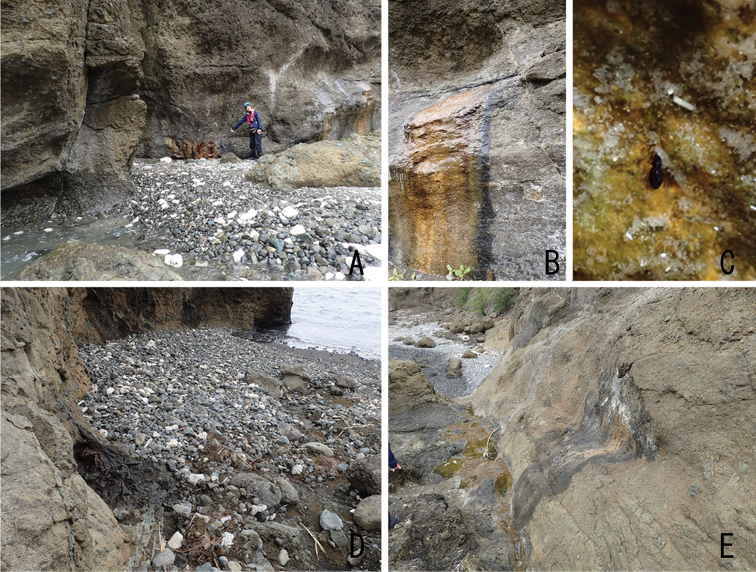
Habitat of *Ochthebiussasakii* sp. n. **A–C** Chichi-jima **D** Otouto-jima **E** Ani-jima.

Figs 1 , 2 , 3 , 4 , 5 Japanese name: Ogasawara-sesuji-darumagamushi

#### Type series.

**Holotype** (EUMJ): male, “Higashi-kaigan, Chichi-jima, Ogasawara Isls., Japan, 22.II.2018, H. Karube leg.”. **Paratypes** (EUMJ, NMW, KPMNH): 5 exs, same data as for the holotype; 10 exs, same locality and collector, but “24.II.2019”; 1 ex., same locality and collector, but “25.II.2019”; 10 exs, “Inui-sawa, Ani-jima, Ogasawara Isls., Japan, 22. II. 2018, H. Karube leg.”; 6 exs, “Kohama, Otouto-jima, Ogasawara Isls., Japan, 22.II.2018, H. Karube leg.”.

#### Diagnosis.

Ochthebius (Ochthebius) sasakii belongs to the *punctatus* species group (sensu Villastrigo et al. 2019) and is similar to two Japanese species, *O.inermis* and *O.danjo*. The adult of *O.sasakii* differs from the two Japanese species in having a smaller body size (see Table 1), anterior margin of labrum not shallowly concave, median groove of pronotum shallow, and short elytral setae. The larva of this species differs in the following characteristics: mandibles strongly curved inwardly in the apical part (gently curved in *O.inermis* and *O.danjo*); labrum arcuate in anterior margin (projecting apically in *O.inermis*); and presence of subprimary setae on pronotum and mesonotum (see Table 2).

**Table 1.** Measurement of three Japanese species of the *Ochthebius*punctatus species group.

	*** O. sasakii ***	*** O. inermis ***	*** O. danjo ***
no.	13	16	6
TL (mm)	1.83–2.16 (2.00)	2.20–2.55 (2.37)	2.42–5.47 (2.99)
HL (mm)	0.30–0.42 (0.39)	0.45–0.52 (0.48)	0.45–0.55 (0.50)
PW (mm)	0.55–0.63 (0.60)	0.67–0.76 (0.70)	0.68–0.74 (0.71)
PL (mm)	0.40–0.48 (0.44)	0.46–0.55 (0.50)	0.50–0.55 (0.54)
EL (mm)	1.03–1.28 (1.17)	1.25–1.50 (1.39)	1.40–4.45 (1.96)
EW (mm)	0.72–0.90 (0.83)	0.45–0.52 (0.48)	0.90–1.00 (0.97)
PW/PL	1.21–1.50 (1.36)	1.34–1.50 (1.40)	1.29–1.36 (1.33)
EL/EW	1.31–1.53 (1.41)	0.35–3.26 (2.32)	1.42–4.49 (2.01)
EL/PL	2.29–2.98 (2.66)	2.60–3.09 (2.81)	2.58–8.09 (3.63)
EW/PW	1.24–1.50 (1.39)	0.61–0.72 (0.68)	1.29–1.41 (1.36)
TL/EW	2.17–2.75 (2.42)	4.64–5.35 (4.98)	2.50–5.53 (3.08)

**Table 2.** Chaetotaxy of 3^rd^ instar larvae of *Ochthebius* spp. (after Delgado and Matsui for *O.danjo*). Cross: present; dash: absent.

		*O.sasakii* sp. nov.	* O. inermis *	* O. danjo *
		present study	present study	Delgado & Matsui 2000
pronotum	A1–A4	×	×	×
	L1–L3	×	×	×
	P1–P4	×	×	×
	Da1, Db1, Dc1	×	×	×
	C1	×	×	×
	C2, C3	–	×	×
	C4, C5	×	×	×
	Da'–Dc', Dc''	×	–	–
mesonotum	A1–A4	×	×	×
	L1–L3	×	×	×
	P1–P4	×	×	×
	Da'	×	–	–
tergum I	A1, A3–A4	×	×	×
	L1–L3	×	×	×
	P1, P3–P4	×	×	×
	P2	×	–	–
	DP1, DP2	×	×	×
	C1–C2, C4	–	–	–
	C3, C5	×	×	×

#### Description.

Adults. Body oblong, slightly convex dorsally, weakly shiny in dorsal surface. Coloration of body black, with weak bluish lustre; ventral surface of body blackish brown; antennae, maxillary palpi and legs yellowish brown, but fuscous in antennomeres IV–IX, terminal palpomere of maxillary palpi and femur.

Head (Fig. 1B) finely punctate, distinctly microreticulate, with deep ocular grooves, closely covered with short setae; fronto-clypeal suture straight. Labrum (Figs 1B, 2C) transverse, finely punctate, almost straight in front margin from dorsal view (Fig. 1B), but shallowly concave from antero-dorsal view (Fig. 2C). Maxillary palpi (Fig. 2B) long, provided with oblong terminal palpomere; approximate ratio of each palpomere (n = 1) as 10 : 14 : 9. Antennae (Fig. 2A) relatively short; approximate ratio of each antennomere (n = 1) as 22.5 : 9.0 : 4.5 : 1.0 : 1.5 : 3.0 : 2.5 : 2.0 : 7.0. Pronotum (Fig. 1C) transversely rectangular, widest at anterior 1/3, finely punctate, distinctly microreticulate, bearing short setae same as in head; anterior margin almost straight, without postocular tooth; posterior margin slightly bisinuous; lateral margins arcuate in anterior parts, straightly tapered in posterior parts; median groove shallow; anterior and posterior discal foveae shallow and indistinct; lateral portions (“ear” in Jäch 1998) slightly depressed dorsally; hyaline membranous cuticula present on anterior and posterior margins; PW/PL 1.21–1.50 (1.36). Elytra oval, gently arcuate in lateral margins, broadest at the middle, irregularly and finely punctate, bearing fine short suberect setae; lateral gutter narrowly explanate; epipleura pubescent, almost reaching elytral apices; elytral apices subacuminate; EL/EW 1.31–1.53 (1.41); EL/PL 2.29–2.98 (2.66); EW/PW 1.24–1.50 (1.39); TL/EW 2.17–2.75 (2.42). Metaventrite pubescent. Legs rather long and slender. Ventrites I–V pubescent; ventrite VI glabrous.

Male. Sternite X (Fig. 2F) subtriangular, with long spiculum. Aedeagus (Fig. 2D, E) ca 0.4 mm, gently curved ventrally; main piece pointed at apex, with three minute setae in subapical area; parameres long, close to main piece, expanded in apical parts.

Female. Sexual dimorphism indistinct in elytral gutter and elytral apices. Capsule of spermathecal duct (Fig. 2G) relatively short.

Measurements (n = 13). TL 1.83–2.16 (2.00) mm; HL 0.30–0.42 (0.39) mm; PW 0.55–0.63 (0.60) mm; PL 0.40–0.48 (0.44) mm; EL 1.03–1.28 (1.17) mm; EW 0.72–0.90 (0.83) mm.

#### Description of third instar larva

(based on a damaged specimen collected with adults from Chichi-jima). Body about 2.0 mm in fully expanded specimen. Coloration of body blackish brown, weakly shining; legs infuscate. Head (Fig. 3A) with five stemmata on each side. Labrum (Fig. 3B) arcuate in anterior margin. Antennae (Fig. 3C) short; IIS1 long, slightly longer than antennomere III; antennomere III about 0.5 times as long as antennomere II. Mandibles (Fig. 3D) strongly curved inwardly in apical parts. Maxillae (Fig. 3E) with palpomere III long and slender. Labium (Fig. 3F) with short mentum. Urogomphi (Fig. 3G) relatively short; URI stout, 4.5 times as long as URII. Chaetotaxy on head capsule, labrum, antennae, maxillae, labium, and urogomphi same as in *O.danjo* (Delgado and Matsui 2000). Pronotum (Fig. 4A) about 1.8 times as wide as long; four anterior (A1–A4), three lateral (L1–L3), four posterior (P1–P4), row of Da1, Db1, and Dc1, four subprimary setae (Da’, Db’, Dc’, Dc’’), and three campaniform sensilla (C1, C4, C5) present on each side. Mesonotum (Fig. 4B) about 2.5 times as wide as long, four anterior (A1–A4), three lateral (L1–L3), four posterior (P1–P4), row of Da1, Db1 and Dc1, subprimary setae (Da’), three campaniform sensilla (C3–C5) present on each side; A4 somewhat longer. Abdominal tergum I (Fig. 4C) about 4.4 times as wide as long, three anterior (A1, A3–A4), three lateral (L1–L3), four posterior (P1–P4), DP1 and DP2, two campaniform sensilla (C3, C5) present on each side.

#### Biological notes.

All the specimens (both adults and larvae) were collected from the surface of littoral rocks covered with a film of sheeting fresh water (depth ca 1–2 mm; Fig. 5). All habitats are situated on the seashore (the nearest point from the edge of the water was ca 3 m); we could not find any habitats in inland areas.

The fauna of the Ogasawara Islands was seriously affected by a long drought in 2016–2017. In addition, a serious drought occurred from autumn to winter 2018/2019. In February 2018, HK found many individuals of *O.sasakii* sp. n. at the Ani-jima site (Fig. 5E) and collected some specimens as the type series. However, in February 2019, HK could not find this species at this site because sheeting fresh water had completely dried up. At the Chichi-jima site, HK found many individuals of this species in both 2018 and 2019, but the area of sheeting fresh water was markedly reduced.

#### Distribution.

Ogasawara Isls. (Chichi-jima, Ani-jima, Otouto-jima).

##### Description of third instar larva of *Ochthebiusinermis*

#### Specimens examined.

20 exs (mature larvae, EUMJ), Shakunouchi-koen, Unnan-shi, Shimane Pref., 24.VI.2006, M. Hayashi leg.

#### Description.

**Figure 6.**
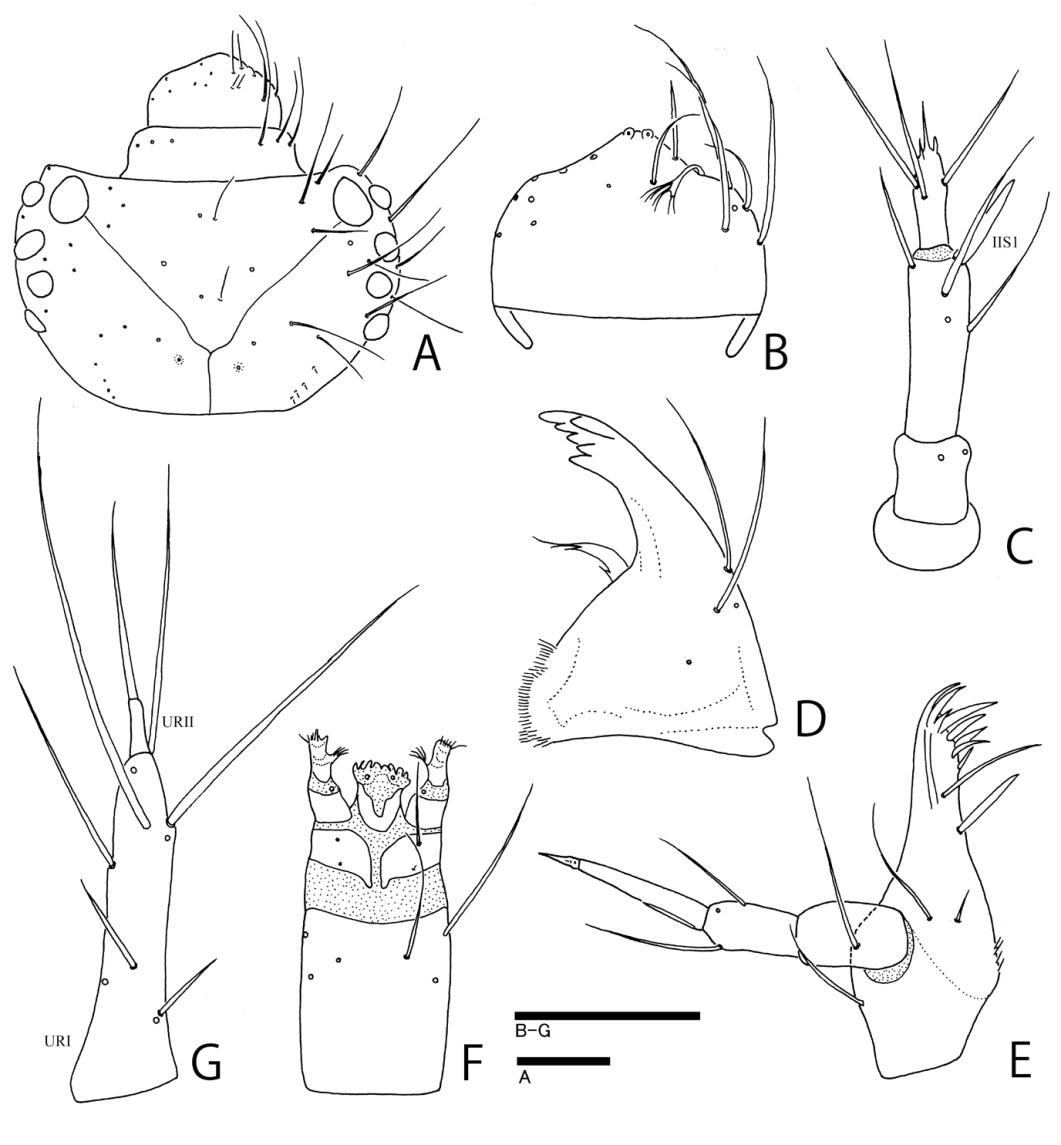
Larva of *Ochthebiusinermis*. **A** head capsule **B** labrum **C** antenna **D** mandible **E** maxilla **F** labium **G** urogomphus. Scale bars: 0.1 mm.

**Figure 7.**
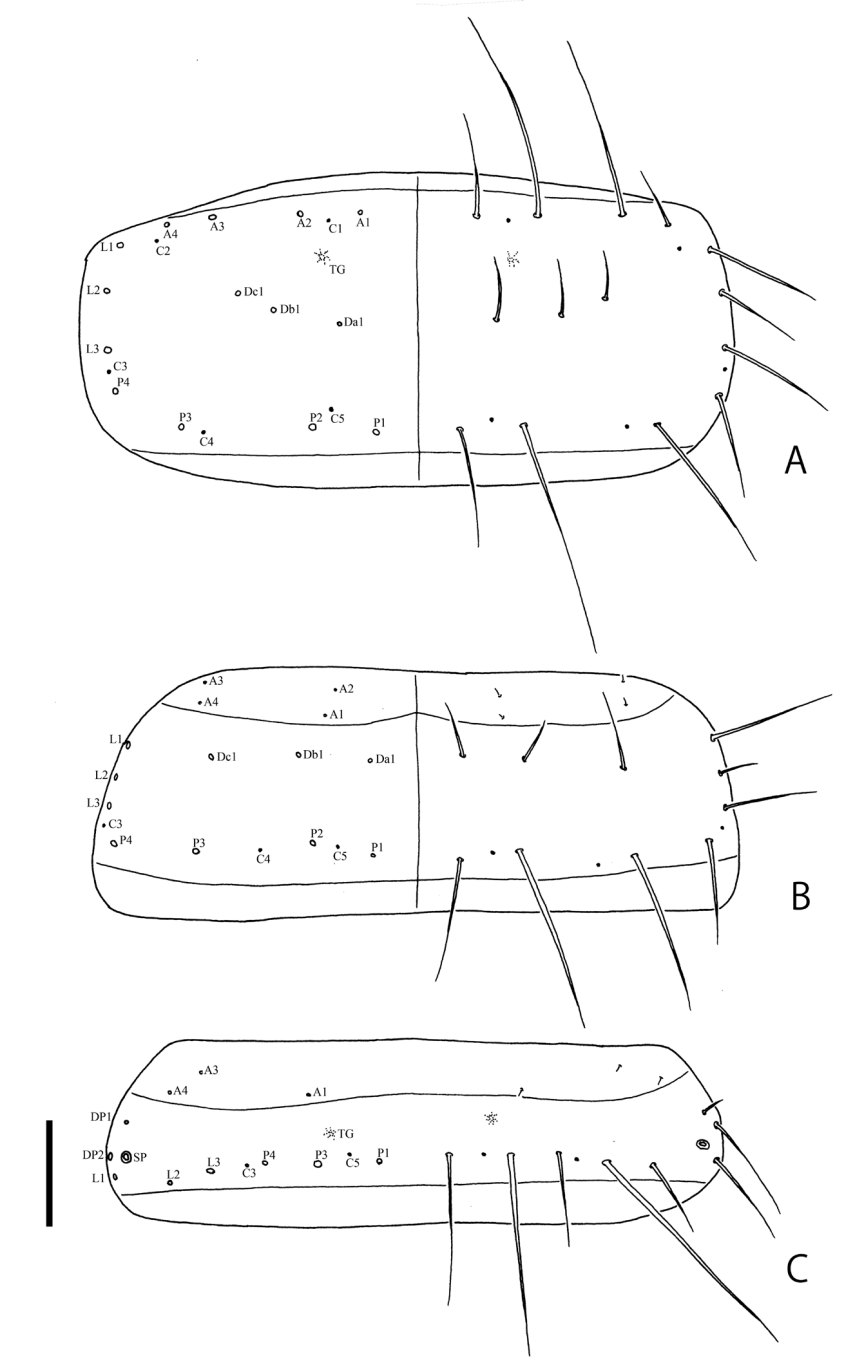
Larval chaetotaxy of *Ochthebiusinermis***A** pronotum **B** mesonotum **C** abdominal tergum I. Scale bar: 0.1 mm.

Body about 3.0 mm in fully expanded specimens. Coloration of body black, strongly shining; legs cream (see Hayashi 2008a: fig. 2F). Head (Fig. 6A) with five stemmata on each side. Labrum (Fig. 6B) projecting apically in anterior margin. Antennae (Fig. 6C) long; IIS1 long, as long as antennomere III; antennomere III long, about 0.6 times as long as antennomere II. Mandibles (Fig. 6D) gently curved inwardly in apical parts. Maxillae (Fig. 6E) with palpomere III long and slender. Labium (Fig. 6F) with long mentum. Urogomphi (Fig. 6G) relatively long; URI slender, about 6.5 times as long as URII. Chaetotaxy on head capsule, labrum, antennae, maxillae, labium, and urogomphi same as in *O.sasakii* sp. n. and *O.danjo* (Delgado and Matsui 2000; present study). Pronotum (Fig. 7A) about 2.0 times as wide as long; four anterior (A1–A4), three lateral (L1–L3), four posterior (P1–P4), row of Da1, Db1 and Dc1, five campaniform sensilla (C1–C5) present on each side. Mesonotum (Fig. 7B) about 2.5 times as wide as long; four anterior (A1–A4), three lateral (L1–L3), four posterior (P1–P4), row of Da1, Db1 and Dc1, three campaniform sensilla (C3–C5) present on each side. Tergum I (Fig. 7C) about 3.3 times as wide as long; three anterior (A1, A3–A4), three lateral (L1–L3), three posterior (P1, P3–P4), DP1, and DP2 setae, two campaniform sensilla (C3, C5) present on each side.

## Discussion

The new species is closely related to two Japanese species, viz., *O.inermis* distributed in Japan (Honshu, Shikoku, Kyushu), Kunashir, and Taiwan, and *O.danjo* distributed in southern Kyushu (including Danjo Islands and Yakushima). The former species inhabits mainly stagnant or flowing water along rivers, usually associated with filamentous green algae (Jäch 1998), and is sometimes collected from hygropetric microhabitats in mountainous areas (e.g., Yoshitomi 2001) or rocky seashores (e.g., Sugaya 2009). The latter species was found in marine rock pools (Delgado and Matsui 2000; Hayashi 2008b). *Ochthebiussasakii* sp. n. and *O.inermis* live in fresh water, whereas *O.danjo* lives in brackish water.

### Key to species of adult Ochthebius (O.) punctatus species group of Japan

**Table:** 

1	Elytra subparallel-sided, bearing long setae; anterior margin of labrum excised; apex of median piece short (Jäch 1998, fig. 23)	*** O. danjo ***
–	Elytra arcuate laterally, bearing short setae; anterior margin of labrum gently arcuate or almost straight in dorsal view; apex of median piece long	**2**
2	Body larger (2.2–2.6 mm); anterior margin of labrum gently arcuate; elytral setae longer; median groove of pronotum distinct; median piece strongly curved; distal lobe expanded apically (Jäch 1998, fig. 22)	*** O. inermis ***
–	Body smaller (1.8–2.2 mm); anterior margin of labrum shallowly concave in antero-dorsal view; elytral setae shorter; median groove of pronotum shallow; median piece gently curved; distal lobe slender (Fig. 2D, E)	***O.sasakii* sp. n.**

## Supplementary Material

XML Treatment for Ochthebius (Ochthebius) sasakii
